# NAD^+ ^metabolite levels as a function of vitamins and calorie restriction: evidence for different mechanisms of longevity

**DOI:** 10.1186/1472-6769-10-2

**Published:** 2010-02-22

**Authors:** Charles Evans, Katrina L Bogan, Peng Song, Charles F Burant, Robert T Kennedy, Charles Brenner

**Affiliations:** 1 Department of Chemistry, University of Michigan, Ann Arbor, MI 48109, USA; 2 Michigan Metabolomics and Obesity Center, University of Michigan, Ann Arbor, MI 48109, USA; 3 Biochemistry Graduate Program, Dartmouth Medical School, Lebanon, NH 03756, USA; 4 Department of Biochemistry, Carver College of Medicine, University of Iowa, Iowa City, IA 52242, USA; 5 Department of Chemistry, University of Michigan, 930 N University Ave, Ann Arbor, MI 48109, USA; 6 Department of Biochemistry, Carver College of Medicine, University of Iowa, 51 Newton Rd, 4-403 BSB, Iowa City, IA 52242, USA

## Abstract

**Background:**

NAD^+ ^is a coenzyme for hydride transfer enzymes and a substrate for sirtuins and other NAD^+^-dependent ADPribose transfer enzymes. In wild-type *Saccharomyces cerevisiae*, calorie restriction accomplished by glucose limitation extends replicative lifespan in a manner that depends on Sir2 and the NAD^+ ^salvage enzymes, nicotinic acid phosphoribosyl transferase and nicotinamidase. Though alterations in the NAD^+ ^to nicotinamide ratio and the NAD^+ ^to NADH ratio are anticipated by models to account for the effects of calorie restriction, the nature of a putative change in NAD^+ ^metabolism requires analytical definition and quantification of the key metabolites.

**Results:**

Hydrophilic interaction chromatography followed by tandem electrospray mass spectrometry were used to identify the 12 compounds that constitute the core NAD^+ ^metabolome and 6 related nucleosides and nucleotides. Whereas yeast extract and nicotinic acid increase net NAD^+ ^synthesis in a manner that can account for extended lifespan, glucose restriction does not alter NAD^+ ^or nicotinamide levels in ways that would increase Sir2 activity.

**Conclusions:**

The results constrain the possible mechanisms by which calorie restriction may regulate Sir2 and suggest that provision of vitamins and calorie restriction extend lifespan by different mechanisms.

## Background

**Figure 1:**
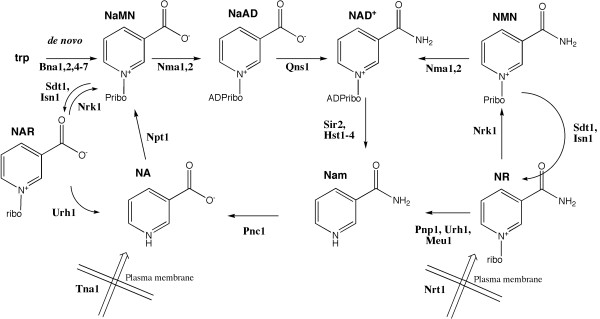
***De novo *synthesis begins with tryptophan, which is converted to NaMN in six enzymatic steps**. NaMN is then adenylylated by Nma1 and Nma2 to NaAD, which is converted to NAD^+ ^by glutamine-dependent NAD^+ ^synthetase, Qns1. NaMN is also formed by salvage of Nam, *via *nicotinamidase, Pnc1, and nicotinic acid phosphoribosyltransferase, Npt1. Environmental NA also generates NaMN *via *the phosphoribosylation activity of Npt1. NR is converted to NMN *via *Nrk1, then to NAD^+ ^by Nma1 and Nma2. Additionally, NAR can utilize Nrk1-dependent phosphorylation and both NR and NAR can be converted to NAD^+ ^in Nrk1-independent pathways initiated by nucleoside splitting activities. The nucleosides NR and NAR are produced intracellularly by the 5'-nucleotidase activities of Sdt1 and Isn1 acting on NMN and NAR. NAD^+ ^is broken down by the sirtuins, Sir2 and Hst1-4.

The pyridine dinucleotide, NAD^+^, synthesized from the pyridine mononucleotides nicotinic acid mononucleotide (NaMN) or nicotinamide mononucleotide (NMN), is a unique cellular molecule that cycles between NAD^+ ^and NADH co-enzymatically and is cleaved to produce nicotinamide (Nam) and acetylated ADPribose in protein lysine deacetylation by sirtuins [1]. Typically, eukaryotic *de novo *biosynthetic pathways produce NaMN from tryptophan through quinolinic acid (QA) and from the vitamin, nicotinic acid (NA). Eukaryotic salvage pathways produce NMN from Nam and/or nicotinamide riboside (NR) [2,3]. The biosynthetic pathways for NAD^+ ^in *Saccharomyces cerevisiae *are schematized in Figure 1. Though most eukaryotic NAD^+ ^biosynthetic pathways were described long ago, we have only recently determined the gene and enzyme basis for the NR kinase pathway [4], NR kinase-independent NR salvage [5,6], specific NR transport [7], specific utilization of nicotinic acid riboside (NAR) [8,9], and dephosphorylation of NMN and NaMN to NR and NAR [10].

Several lines of reasoning indicate that the availability of salvageable vitamin precursors and the regulation of salvage enzymes are key determinants of Sir2 function. First, the NA salvage enzyme nicotinic acid phosphoribosyl transferase, Npt1, is required for the longevity benefit of calorie restriction (CR) [11]. Second, the Nam salvage enzyme nicotinamidase, Pnc1, is induced by CR and other stresses and its induction correlates with increased lifespan [12]. Third, yeast cells grown without NA or NR are fully viable but have a short replicative lifespan [5]. Fourth, provision of NR extends lifespan in a manner that depends on NR salvage enzymes, Nrk1, Urh1 and Pnp1 [5]. However, there have been fundamental critiques of the body of work that has attributed the longevity benefit of CR to Sir2. In particular, lifespan extension independent of Sir2 has been demonstrated and a Sir2-independent target of nicotinamide has been proposed [13]. Additionally, the only report that Sir2 exhibits increased gene silencing activity upon CR [14] appears to be artifactual [15,16]. Though Sir2 may not increase gene silencing upon CR, it appears that CR results in reduced ribosomal DNA recombination via a mechanism involving reduced Tor signaling [15,17] and/or the association of Sir2 with ribosomal genes at the inner nuclear membrane [16,18].

Several models have been developed to account for how NAD^+ ^metabolism might be regulated by CR in a manner that would increase Sir2 localization and/or function. According to one model, CR lowers Nam levels, thereby relieving Sir2 inhibition [12]. A second model claims that CR lowers NADH levels, thereby relieving Sir2 competitive inhibition [19]. Our prior work indicates that increased net NAD^+ ^synthesis in the presence of salvageable vitamins accounts for increased Sir2 function [5], though we had not determined whether NAD^+ ^metabolites are altered by CR. Moreover, our recent work indicates that Isn1, which encodes an IMP, NMN and NaMN 5'-nucleotidase activity, is increased in expression by provision of glucose and nicotinic acid [10]. Here, to dissect the basis for longer lifespan in vitamin-replete versus vitamin-free medium and to determine whether NAD^+ ^metabolism is altered by CR, we developed a liquid chromatrography mass spectrometry (LC-MS) method to analyze the yeast NAD^+ ^metabolome. The method, utilizing electrospray ionization (ESI) and selected reaction monitoring (SRM), is powerful and sensitive, providing detection of all metabolites, including the predicted metabolites, NAR and NR. The data indicate that increased net NAD^+ ^synthesis in rich and vitamin-replete medium may explain increased lifespan with respect to vitamin-free medium. However, the data are largely inconsistent with levels or ratios of NAD^+^, Nam or NADH as mediators of the effect of glucose limitation on lifespan, suggesting that provision of vitamins and CR extend lifespan by different mechanisms. Moreover, the levels of NaMN and NMN are sufficient to allow regulated expression of NaMN/NMN adenylyltransferases and NaMN/NMN 5' nucleotidases [10] to push NAD^+ ^metabolism forward to NaAD and NAD^+ ^or backward to NAR and NR.

## Results and Discussion

### Identification of the core NAD+ metabolome in extracts of ml cultures of yeast

**Figure 2:**
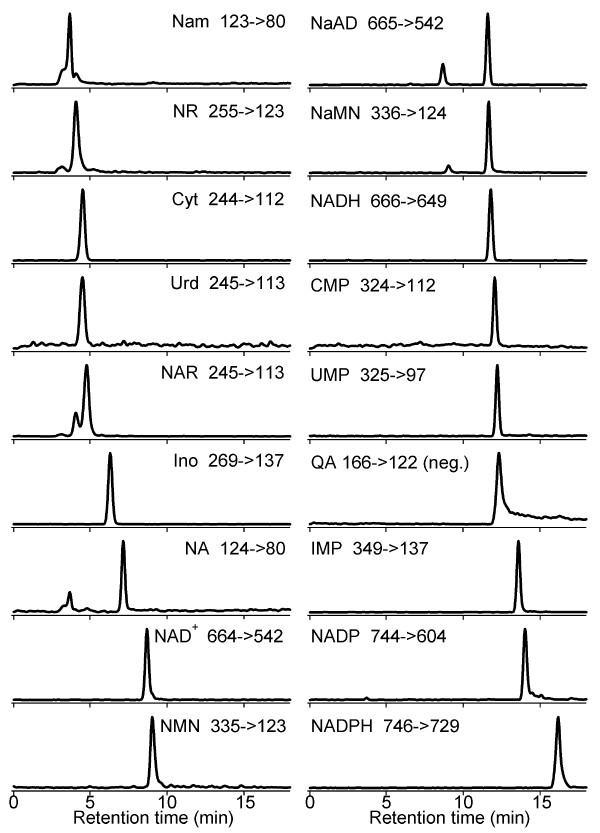
**Chromatograms of 18 NAD**^**+**^**-related compounds generated using SRM LC-MS**. All compounds except quinolinic acid were detected using positive ion mode. Injected amount was 10 pmol for all compounds.

Recently, Tsuchiya and co-workers reported an LC-MS method with partial coverage of the NAD^+ ^metabolome. Levels of NAD^+^, NMN, NaMN, ADPribose and AMP were reported in mouse erythrocytes. However, NADH, NADP, NADPH, QA, NR, NAR, NA, Nam, and nicotinic acid adenine dinucleotide (NaAD) were undetected or below detection limits in the biological sample [20]. Lu and co-workers detected NAR and NR in yeast extracts by LC-MS but determined NAD^+ ^levels using a cycling assay [21]. Here, the core NAD^+ ^metabolome was detected and quantified by LC-MS using 1% of the yeast cell number of the previous method. Keys to the present method were HILIC on an aminopropyl stationary phase at basic pH, positive ion mode SRM detection of 17 compounds, and negative ion mode detection of QA. Separation and fragmentation behaviors of the 18 compounds are shown in Figure 2. As shown in Table 1, the limits of quantification (LOQ) for 18 metabolites were in a 20-fold range from 0.06 pmol (NAR and NADH) to 1.2 pmol (NA and Urd). Technical replication errors measured in relative standard deviation (RSD) were 10% or less for 16 of 18 compounds.

**Table 1:** LC-MS/MS SRM parameters and sensitivity details for NAD^+ ^metabolites and related nucleosides and nucleotides

Metabolite	Parent mass	Product mass	Cone voltage	Collision Energy	RT (min)	LOQ (pmol)	**R**^**2**^	RSD (%)
Nam	123	80	35	20	3.68	0.45	0.996	7
NA	124	80	35	20	7.15	1.20	0.999	12
Cyt	244	112	35	10	4.52	0.10	0.995	10
Urd	245	113	90	10	4.56	1.20	0.978	10
NR	255	123	50	10	4.07	0.20	0.992	7
NAR	256	124	35	10	4.80	0.06	0.984	5
Ino	269	137	80	10	6.31	0.07	0.993	3
CMP	324	112	35	10	12.05	0.68	0.999	6
UMP	325	97	90	15	12.22	0.21	0.989	8
NMN	335	123	35	10	9.04	0.50	0.988	9
QA	166 (-)	122	35	10	12.34			9
NaMN	336	124	35	10	11.66	0.18	0.999	5
IMP	349	137	80	10	13.59	0.13	0.997	5
NAD^+^	664	542	35	18	8.69	0.17	0.996	8
NaAD	665	542	35	20	11.59	0.26	0.987	8
NADH	666	649	35	20	11.77	0.06	0.991	16
NADP	744	604	35	25	14.01	0.87	0.995	9
NADPH	746	729	35	20	16.18	0.17	0.994	8

To determine whether metabolite standards are stable in extraction conditions, triplicate mixtures of authentic NAD metabolite standards were placed into to 0.3 mL of boiling buffered ethanol and maintained at 80°C for 3 minutes. For comparison, identical mixtures of NAD metabolite standards were combined with 0.3 mL cold buffered ethanol and were maintained on ice. Both groups of samples were dried by Speedvac and reconstituted in 80% methanol/20% water, and were then analyzed by LC-MS. For all metabolites, average decreases in metabolite peak area from exposure to boiling extraction solvent were determined to be 15% or less. The observed decreases were not found to be statistically significant for any compound except inosine, which showed a 13.9% decrease (p = 0.04). These data are shown in Additional file 1, Table S1.

To validate the quantitative accuracy of the method, it was necessary to determine whether ionization suppression and other matrix effects might affect the validity of metabolite calibration curves in the presence of cellular extracts. To develop an extract nearly devoid of the metabolites in question, an isotopically labeled yeast extract was prepared by growing Fleischmann's yeast on medium containing uniformly ^13^C-labeled glucose (97%). The ^13^C yeast cultures were harvested at the same optical density as the unlabeled yeast cultures, and metabolites were extracted from ^13^C-labeled yeast using the boiling buffered ethanol procedure [22]. The extract was then spiked with 5 μM ^12^C authentic NAD metabolite standards and analyzed using LC-MS/MS.

To assess ionization suppression, the peak areas for the ^12^C standards in the ^13^C yeast extract were compared to the peak areas for a pure ^12^C standard mixture. These data are shown in Additional file 1, Table S2. All but three of the compounds showed no to moderate (<30%) reduction in signal in the presence of ^13^C yeast extract. Thus, for the majority of the components of the NAD^+ ^metabolome, the method is sufficiently free from ionization suppression to allow direct quantification of the components of the NAD^+ ^metabolome in biological extracts. The three compounds that showed a more severe reduction of signal in the ^13^C yeast extract were NAD^+ ^(49% attenuation of signal), Ino (70% attenuation), and NA (76% attenuation). For these compounds, determination of absolute concentrations of the metabolites in cell extracts is subject to larger errors, although relative quantification is still possible. More robust quantification could be achieved with isotopically labeled internal standards for those components of the NAD^+ ^metabolome prone to ionization suppression, or by inclusion of a global internal standard, such as an isotopically labeled extract, in all samples.

### Increased net NAD+ biosynthesis in rich and vitamin-supplemented media

Yeast cells are either grown in rich medium, containing yeast extract, or in synthetic medium, which contains salts, metals, specific amino acids and nucleobases, and a selection of vitamins [23]. Because yeast nitrogen base was formulated to support the growth of every yeast species [24] including *Candida glabrata*, which has no *de novo *biosynthetic capacity [25], synthetic yeast medium typically contains 3 μM NA. NA-free synthetic medium supports the growth of wild-type *S. cerevisiae *but the replicative lifespan on such medium is approximately half that of cells grown on SDC with NA, SDC with NR or on YPD [5]. Earlier, we reported that yeast cells grown on SDC with NA have an intracellular NAD^+ ^concentration of ~2 mM, which declines to about 1 mM as cells reach stationary phase, that yeast cells grown on vitamin-free SDC have an intracellular NAD^+ ^concentration of ~1 mM, and that yeast cells grown on SDC with 10 μM NR in place of NA have a stable intracellular NAD^+ ^concentration of ~2 mM. To test whether the LC-MS method could detect two-fold differences in NAD^+ ^levels and to determine whether other metabolites are altered by medium components other than glucose, we subjected cells grown in YPD, SDC and SDC without NA to extraction and metabolite analysis by LC-MS. As shown in Table 2, the availability of yeast extract and NA clearly increases net NAD^+ ^synthesis by two-fold into the NAD^+ ^and NADH pools, consistent with the increased Sir2 functions and replicative longevity of cells grown in such conditions [5].

**Table 2:** Intracellular micromolar concentrations of NAD^+ ^metabolites and related nucleosides and nucleotides as a function of medium components

Metabolite	SDC-NA	SDC	YPD	YPD0.5	YPD0.2
Nam	1.5 ± 0.2*†	31.6 ± 3.0	35.0 ± 3.2	29.7 ± 0.3	51.9 ± 17.9
NA	<0.5	<0.5	<0.5	<0.5	<0.5
Cyt	1.8 ± 0.2†	1.5 ± 0.2†	69.5 ± 11.5	42.2 ± 9.1	63.4 ± 13.5
Urd	5.3 ± 1.4†	13.0 ± 3.0†	139.7 ± 0.6	141.7 ± 0.2	128.7 ± 7.0
NR	2.1 ± 0.3*†	4.6 ± 0.9†	9.3 ± 0.4	7.9 ± 1.5	7.1 ± 1.4
NAR	0.2 ± 0.0*†	8.1 ± 1.3	12.5 ± 0.2	8.3 ± 1.7	8.4 ± 0.7†
Ino	21.8 ± 1.7*†	6.0 ± 0.4†	54.9 ± 2.6	38.5 ± 3.3†	31.8 ± 3.3†
CMP	31.5 ± 1.5	39.7 ± 9.1	146.4 ± 16.7	134.8 ± 45.1	165.3 ± 38.3
UMP	63.0 ± 7.0*†	145.4 ± 27.9	179.6 ± 2.7	238.0 ± 20.9	236.6 ± 23.2
NMN	19.0 ± 1.9*†	67.3 ± 13.0	62.1 ± 13.7	60.0 ± 14.8	72.4 ± 21.2
NaMN	2.9 ± 0.2*†	28.0 ± 4.5	11.6 ± 2.0	18.4 ± 4.3	21.1 ± 3.7
IMP	8.4 ± 0.3	7.1 ± 1.8	16.8 ± 6.3	16.1 ± 1.5	16.1 ± 1.5
NAD^+^	277.2 ± 26.7*	513.9 ± 51.1	438.5 ± 66.7	308.9 ± 33.8	339.5 ± 11.7
NaAD	<0.1	<0.1†	3.8 ± 0.3	<0.1†	<0.1†
NADH	125.8 ± 13.6*†	207.3 ± 17.1	263.0 ± 55.3	101.2 ± 24.6	152.0 ± 12.7
NADP	31.2 ± 1.4	31.1 ± 3.5	46.9 ± 10.2	42.9 ± 7.2	52.5 ± 2.1
NADPH	0.3 ± 0.1*	3.1 ± 0.6	0.7 ± 0.2	1.3 ± 0.1	3.0 ± 0.2†‡

* indicates a significant difference when compared to cells grown in SDC. † indicates a significant difference when compared to cells grown in YPD. ‡ indicates a significant difference between cells grown in YPD 0.5 and YPD 0.2% glucose.

Because prior investigations of the effect of NA, NR and NAR only examined accumulation of NAD^+ ^[5,8], it was interesting to examine how provision of NA or yeast extract and peptone (i.e., YPD) might alter the NAD^+ ^metabolome as a whole. As shown in Table 2, NAD^+ ^metabolites fall into several categories with respect to provision of NA and yeast extract. Whereas NAD^+ ^and NADH increase approximately two-fold with either life-extending change in medium, NADP and NADPH are little changed. In contrast, the intracellular levels of NR and NMN were increased 2-4 fold, NaMN 4-10 fold, and Nam and NAR increased >20-fold by both life-extending conditions. These data suggest that not only is net NAD^+ ^synthesis increased by medium components, but that NAD^+ ^anabolic and catabolic processes are quantitatively and/or qualitatively altered by provision of medium components.

In addition to NAD^+ ^metabolites, we measured three nucleosides (Urd, Cyt and Ino) and three nucleoside monophosphates (UMP, CMP and IMP). Each nucleoside was strikingly increased in rich medium, suggesting that these nucleosides are present in yeast extract. Not surprisingly, the corresponding nucleoside monophosphates were also greatly increased by provision of rich medium. For unknown reasons, NA increased the net synthesis of UMP by more than two-fold.

### Glucose limitation only mildly alters levels of NAD+ metabolites

In mouse liver, a one day fast has been reported to elevate NAD^+ ^levels [26]. Similarly, CR has been reported to elevate NAD^+ ^levels in brain [27]. This could either be mediated by increased net NAD^+ ^biosynthesis in these tissues or by increased mobilization of tryptophan or vitamin precursors of NAD^+ ^[2]. To test whether yeast cells have a cell-autonomous mechanism to alter NAD^+ ^levels, we grew wild-type yeast cells in SDC media differing in glucose concentration (2%, 0.5% and 0.2%). Extraction and LC-MS analysis of these samples revealed the apparent intracellular metabolite concentrations shown in Table 2. The results are rather striking for the constancy in metabolite levels as a function of glucose limitation. Excluding NA and NaAD, which were not consistently detected in yeast samples, and NADPH, which was present at <1 μM in the YPD sample, no compound was increased or decreased two-fold by glucose limitation to both 0.5% and 0.2%.

Nam, a sirtuin-inhibiting compound [28], which is present in SDC and YPD samples at ~30 μM and can be detected in SDC -NA samples at 5% of this concentration, was not reduced by glucose limitation. NAD^+^, which was increased by provision of NA or rich medium (life-extending conditions), was *moderately reduced *by glucose limitation, also a life-extending condition. NADH, which was increased by provision of NA and by rich medium, was reduced somewhat more than was NAD^+^. With glucose limitation to 0.5%, NADH was reduced 2.6-fold. With glucose limitation to 0.2%, NADH was reduced 1.7-fold. In contrast, the apparent levels of NADPH were increased two-fold and four-fold by glucose limitation to 0.5% and 0.2%, respectively, though the absolute contribution of the NADPH fraction of pyridine dinucleotides remained very small. Such data suggest the possibility that an NADPH-generating process may be positively regulated by CR, though the target or the significance of elevated NADPH in replicative longevity is unknown.

### Levels and ratios of NAD+ metabolites

The present study indicates that yeast cells increase net NAD^+ ^biosynthesis with additions of yeast extract and NAD^+ ^precursor vitamins to the medium. Our previous work suggests that yeast cells relying on *de novo *NAD^+ ^biosynthesis have an intracellular NAD^+ ^concentration of ~1 mM that satisfies vital requirements but provides little support for Sir2 function. According to these data, intracellular NAD^+ ^concentrations above ~1 mM are associated with longer replicative lifespan and cellular NAD^+ ^concentrations vary from ~1 mM to ~2 mM [5]. By LC-MS, the NAD^+ ^concentration is ~0.28 mM in vitamin-free medium and twice that when cells are grown with NA. The lower NAD^+ ^concentrations in LC-MS measurements are likely a result of ion suppression.

With the *caveat *that ionization suppression reduced the observed levels of NAD^+ ^more than NADP, NADH and NADPH, it is possible to use the NAD^+ ^metabolomic data collected herein to compare the ratios of the four major co-enzymatic forms of NAD^+ ^and determine whether such ratios are altered by medium components including NA and glucose. As shown in Table 3, NADH as a proportion of NAD^+ ^is higher for cells grown in YPD than in the other conditions. Consistent with a model of CR that glucose limitation would create a more oxidizing environment with a higher NAD^+^:NADH ratio [19], cells grown in 0.5% glucose had a higher NAD^+^:NADH ratio than cells grown in 2% glucose. However, because NADH is such a poor inhibitor of Sir2 (*K*_*i *_~ 17 mM) [29], it is not clear how reducing NADH concentrations from ~0.26 mM to the 0.10 to 0.15 mM range would increase Sir2 activity. Similarly, it remains to be seen if the increased NADPH as a proportion of NAD^+^, which correlated with glucose limitation, is functionally related to the longevity-promoting effects of CR.

**Table 3:** Ratios of key metabolites: dinucleotide subprofile

	NADH	**NAD**^+^	NADP	NADPH
SDC NA	0.45	1.0	0.11	0.001
SDC	0.40	1.0	0.06	0.006
YPD	0.60	1.0	0.11	0.002
YPD 0.5	0.33	1.0	0.14	0.004
YPD 0.2	0.45	1.0	0.15	0.009

Nam is a known product inhibitor of sirtuins [28] and Pnc1, encoding nicotinamidase, is induced by CR conditions and required for the longevity benefit of CR [12]. Thus, the proposed ability of CR to reduce cellular Nam concentrations or increase the NAD^+^:Nam ratio has been an appealing model [12]. However, as shown in Table 2 and Table 4, CR does not reduce Nam concentrations. Consistent with data obtained by ^13^C NMR [30], NAD^+ ^is actually reduced by CR conditions. Thus, as illustrated in Table 4, the ratio of NAD^+ ^to Nam is uncorrelated with increased lifespan. Vitamin-free synthetic medium, which provides the shortest lifespan, had the highest NAD^+^:Nam ratio and reduction of glucose from rich medium reduced the NAD^+^:Nam ratio further.

**Table 4:** Ratios of key metabolites: Nam and NAD^+ ^subprofile

	**NAD**^+^	Nam
SDC -NA	184.	1.0
SDC	16.3	1.0
YPD	12.5	1.0
YPD 0.5	10.4	1.0
YPD 0.2	6.5	1.0

The levels of the pyridine mononucleotides NaMN and NMN as compared to the levels of the dinucleotides NaAD and NAD^+ ^are of interest in two respects. First, the human NaMN/NMN adenylyltransferases are differentially expressed and localized, suggesting that the adenylylation steps in NAD^+ ^synthesis may be points of regulation [31,32]. Second, with our recent discovery of NR as a vitamin and both NR and NAR as apparent yeast metabolites [4,5,8,9], we considered that production of these two nucleosides might be controlled by 5' nucleotidase activities acting on NMN and NaMN and identified Isn1 and Sdt1 as multifunctional 5'-nucleotidases that are responsible for cellular generation of NR and NAR [10]. For the adenylylation steps in NAD^+ ^biosynthesis to be regulated and for production of NR and NAR from NMN and NaMN to be plausible, cells would have to have reasonable concentrations of the pyridine mononucleotides. Though the NaMN/NMN adenylyltransferases are known to perform reversible reactions *in vitro *[33], such reactions, which produce inorganic pyrophosphate, would be rendered irreversibly forward in the direction of dinucleotides if the pyrophosphate is always rapidly consumed to orthophosphate *in vivo*. Though pyrophosphate consumption is dogma, actual measurements of pyrophosphate in budding yeast cells indicate that levels can range from 10-1000 times higher that ATP under various growth conditions [34]. As shown in Table 5, there was 7-21% as much NMN to NAD^+ ^under all conditions examined. Thus, steady state levels of the substrates of NaMN/NMN adenylyltransferases were detected under all conditions measured. In contrast, NaAD, the product of NaMN adenylylation and the substrate of glutamine-dependent NAD^+ ^synthetase Qns1 [35], was below 1% of the level of NAD^+ ^under all conditions examined, suggesting that NaAD produced from the *de novo *or Preiss-Handler pathways is driven forward under *k*_*cat*_/*K*_*m *_conditions for Qns1 [36].

**Table 5:** Ratios of key metabolites: adenylyltransferase subprofile

	NaMN	NaAD	**NAD**^+^	NMN
SDC -NA	0.01	N.D.	1.0	0.07
SDC	0.05	N.D.	1.0	0.13
YPD	0.03	0.009	1.0	0.14
YPD 0.5	0.06	N.D.	1.0	0.19
YPD 0.2	0.06	N.D.	1.0	0.21

Only a few years ago, NMN and NR were not depicted on the metabolic schemes for how yeast cells make NAD^+ ^[12,29,37-39]. As shown in Table 6, NMN is more abundant than NaMN under all conditions examined even though no condition investigated the provision of NR, such that all NAD^+ ^biosynthesis is proceeding through NaMN. Moreover, just as the proportion of NMN to NAD^+ ^suggested that NMN adenylyltransferase is not running irreversibly forward, the proportion of NR and NAR nucleosides to the corresponding NMN and NaMN nucleotides, shown in Table 3D, suggests mechanisms by which the cell may maintain nucleoside pools. Indeed, we have now identified Isn1, previously reported to be an IMP-specific 5'-nucleotidase [40], and Sdt1, previously reported to be a pyrimidine-specific 5'-nucleotidase [41], as responsible for NR and NAR production *in vivo *[10].

**Table 6:** Ratios of key metabolites: nucleoside/nucleotide subprofile

	NAR	NaMN	NMN	NR
SDC -NA	0.01	0.15	1.0	0.11
SDC	0.12	0.42	1.0	0.07
YPD	0.20	0.19	1.0	0.15
YPD 0.5	0.14	0.31	1.0	0.13
YPD 0.2	0.12	0.29	1.0	0.10

## Conclusions

The five growth conditions in this study allow two longevity-promoting regimens to be compared metabolically. Cells grown with yeast extract or nicotinic acid have a longer lifespan than cells grown in vitamin-free synthetic media [5] and cells grown in reduced glucose have a longer lifespan than cells grown in 2% glucose [11]. Eight metabolites (Nam, NR, NAR, UMP, NMN, NaMN, NAD^+ ^and NADH) are statistically significantly higher in both SDC and YPD than in SDC -NA. However, none of these metabolites are higher in both 0.5% and 0.2% glucose than in 2% glucose. Five metabolites are lower in both CR conditions (NAR, Ino, NAD^+^, NaAD and NADH). Strikingly, three of the compounds that rise with provision of vitamins (NAR, NAD^+ ^and NADH) fall in CR conditions. These data suggest that provision of vitamins and CR alter NAD^+ ^metabolism in two different ways and that vitamins increase net NAD^+ ^synthesis in a manner that extends lifespan, whereas CR employs a different mechanism.

The early models of CR either invoked increased flux [42] or simple regulation of a single key Sir2-inhibitory metabolite [19,30]. Though flux has not yet been investigated and the data are inconsistent with simple regulation of NAD^+^, NADH or Nam, it remains to be determined whether the NAD^+ ^metabolite profile is regulated in a complex manner by CR in a manner that determines Sir2 biological activity or whether the metabolic effects of CR are largely mediated in a Sir2-independent manner. Models that depend on increased flux will almost certainly depend on increased expression of Pnc1, which is induced by CR [12]. Such a phenomenon would seem to be required for Nam salvage to keep up with increased Sir2 activity under CR conditions. However, because Nam levels are not reduced by CR, it is not obvious how Nam clearance can be the driver of increased Sir2 activity.

The NAD^+ ^metabolomic assay described in this study should be empowering for multiple lines of investigation. This study, in addition to corroborating the existence of NAR and NR in cells grown without these precursors [21], demonstrates that yeast cells maintain a substantial proportion of potential NAD^+ ^equivalents in two mononucleotide pools that can be driven forward to produce NaAD and NAD^+ ^or driven backward by dephosphorylation to produce NAR and NR. Such steps in NAD^+ ^metabolism are almost certainly regulated by novel mechanisms. Indeed, the quantitative analysis of NAR, NR and NMN levels in yeast cells supplemented by none of these precursors creates problems that will only be solved by characterization of new genes, enzymes and pathways. With knowledge of the gene set, ongoing studies are designed to determine the rates of flux of metabolites upon imposition of CR and the nature of the nuclear, mitochondrial and cytoplasmic NAD^+ ^metabolomes.

## Methods

### Materials

NR and NAR were synthesized as described [9]. All other NAD^+ ^metabolites, nucleotides, nucleosides, 8-aminooctanoic acid, ammonium acetate, glucose and ammonium hydroxide were purchased from Sigma-Aldrich Corp (St. Louis, MO). HPLC-grade water, acetonitrile and methanol were purchased from Burdick and Jackson (Muskegon, MI).

### Saccharomyces cerevisiae strains, media and extraction

The analyzed yeast strain was BY4742 (*MATα his3Δ1 leu2Δ0 lys2Δ0 ura3Δ0*). The yeast strain grown in ^13^C glucose was Fleischmann's. Cells were grown in synthetic dextrose complete (SDC) medium (1.7 g Difco yeast nitrogen base without (NH_4_)_2_SO_4 _and without amino acids), 5 g of (NH_4_)_2_SO_4_, 0.89 g amino acid mix and 20 g dextrose per liter). SDC -NA contained 6.9 g Formedium (Hunstanton, England) yeast nitrogen base without amino acids and NA, 0.89 g amino acid mix, and 20 g dextrose per liter. YPD contained 10 g yeast extract, 20 g peptone and 20 g dextrose per liter. Experimental cultures were grown overnight, diluted to OD_600 nm _= 0.2, grown to mid-log phase (OD_600 nm _= 0.7-0.8), and collected by centrifugation. Pellets corresponding to 25 ml of culture grown to mid-log phase were collected and stored at -80°C prior to extraction in 0.3 ml of boiling, buffered ethanol (75% ethanol and 25% 10 mM HEPES adjusted to pH 7.1 with ammonium hydroxide), drying by Speedvac, and resuspension in 0.1 ml of water [22]. Samples were then checked by absorbance at 260 nm, allowing the extracts from slightly more dense cultures to be diluted to the same OD_260 nm _as those from less dense cultures. Prior to LC-MS analysis, samples were further diluted 4:1 with methanol to give a final solvent composition of 80% methanol/20% water in order to ensure adequate retention of the metabolites of interest on the HILIC column. Total cell number as a function of cultural density was determined as described [43]. Mol of a metabolite was converted to M units using 7 × 10^-14 ^L as the intracellular volume of a haploid yeast cell [23]. Though no corrections were applied for potentially different and incomplete extraction efficiencies of the metabolites, every metabolite tested, including NAD^+ ^and NADH, has an extraction efficiency of 91% or greater [22]. All metabolite measurements are reported as means from three independent yeast cultures.

### LC-MS

Chromatographic separation was performed using a Waters Nanoacquity UPLC (Milford, MA). Separations were based on hydrophilic interaction liquid chromatography (HILIC), similar to those that have been described [44]. The 150 mm × 1.0 mm i.d. column contained 3 μm Phenomenex Luna NH_2 _particles. The mobile phases were acetonitrile (A) and 5 mM ammonium acetate adjusted to pH 9.9 with ammonium hydroxide (B). Metabolites were separated with a linear gradient (50 μl/min flow rate) from 30% to 100% (B) over 15 min followed by a 5 min hold at 100% (B). The mobile phase was returned to 30% (B) at 20.1 min and the column was re-equilibrated for 10 min prior to the next run. After completion of a day of runs, the column was flushed with 30 column volumes of deionized water and flushed with isopropanol for storage. MS/MS detection was carried out using a Micromass Quattro Ultima tandem quadrupole MS operated in positive ion SRM mode. The output of the LC column was coupled to the MS using the heated ESI probe. Capillary voltage was +3 kV. Desolvation gas and cone gas flow rates were 420 l/hr and 150 l/hr, respectively. The desolvation heater temperature was 350°C and the source was maintained at 140°C. SRM methods were optimized for each compound by performing direct infusion of a 50 μM standard solution in a solvent of 5 mM ammonium acetate (pH 9.9) and acetonitrile (50:50 v/v) using a syringe pump operated at 10 μl/min. SRM parameters for quinolinic acid (QA), which could not be detected as a positive ion, were optimized in negative ion mode using a capillary voltage of -2.5 kV. Optimal MS/MS transitions, cone voltages and collision energies are shown in Table 1. LC-MS was performed using sequential monitoring of 17 SRM transitions (QA was excluded from analyses of yeast samples). The dwell time for each transition was 100 ms, the inter-channel delay was 10 ms, and the delay between scan cycles was 20 ms. The resulting scan rate was one for each transition every 2.11 seconds. Automated peak detection and integration were used to determine peak area and signal to noise ratio. All peaks were visually inspected to ensure that peaks were properly selected and appropriate bounds were used. Calibration curves were generated using 2.5 μl injections of 0.05, 0.1, 0.3, 1, 3, 10, 30, and 100 μM solutions of authentic standards in 80/20 methanol/water. Weighted linear regressions (1/X) were used to assess the linearity of the method and to calculate the concentrations of metabolites in the yeast extract samples.

## Abbreviations

NaMN: The abbreviations used are nicotinic acid mononucleotide; NMN: nicotinamide mononucleotide; Nam: nicotinamide; QA: quinolinic acid; NA: nicotinic acid; NR: nicotinamide riboside; NAR: nicotinic acid riboside; CR: calorie restriction; LC-MS: liquid chromatography-mass spectrometry; SRM: selected reaction monitoring; ESI: electrospray ionization; NaAD: nicotinic acid adenine dinucleotide; LOQ: limits of quantification; RSD: relative standard deviation.

## Authors' contributions

CB, CE, KLB, CFB and RTK designed the experiments. KLB prepared yeast samples. CE and PS performed mass spectroscopy as advised by CFB and RTK. CB, CFB and RTK provided resources. CE, KLB and CB wrote the paper with advice and review from all authors. All authors read and approved the final manuscript.

## Supplementary Material

Additional file 1 **Supplementary tables**. Tables indicating observed change of standard metabolite peak areas under extraction conditions (Table S1) and ion suppression of standard metabolites in the presence of ^13^C-labeled yeast extract (Table S2) Click here for file
