# Supplementary material for: NAD+ metabolite levels as a function of vitamins and calorie restriction: evidence for different mechanisms of longevity
Source: BMC Chem Biol. 2010 Feb 22;10:2. doi: 10.1186/1472-6769-10-2 (PMC2834649; doi:10.1186/1472-6769-10-2)
Supplement: Additional file 1 — Supplementary tables. Tables indicating observed change of standard metabolite peak areas under extraction conditions (Table S1) and ion suppression of standard metabolites in the presence of 13C-labeled yeast extract (Table S2) [file 1472-6769-10-2-S1.DOC]

**Table S1.** Observed change of standard metabolite peak areas after heating at 80 °C for 3 minutes.

| Metabolite | % change |
| --- | --- |
| Nam | -10% |
| NA | -3% |
| Cyt | 4% |
| Urd | -2% |
| NR | -15% |
| NAR | 0% |
| Ino | -14% * |
| CMP | -7% |
| UMP | -6% |
| NMN | -7% |
| NaMN | -5% |
| IMP | -2% |
| NAD^+^ | -11% |
| NaAD | -5% |
| NADH | -7% |
| NADP | -7% |
| NADPH | -8% |

* indicates a statistically significant difference (p < 0.05)

**Table S2.** Observed attenuation of metabolite peak areas in the presence of ^13^C-labeled yeast extract

| Metabolite | % attenuation |
| --- | --- |
| Nam | 23% |
| NA | 76% |
| Cyt | 5% |
| Urd | 30% |
| NR | 21% |
| NAR | 15% |
| Ino | 70% |
| CMP | none observed |
| UMP | none observed |
| NMN | none observed |
| NaMN | none observed |
| IMP | none observed |
| NAD^+^ | 49% |
| NaAD | 20% |
| NADH | 9% |
| NADP | none observed |
| NADPH | 27% |
